# Attentional Focus and Practice Autonomy Enhance Penalty Kick Accuracy in Soccer

**DOI:** 10.3390/sports13100332

**Published:** 2025-10-01

**Authors:** Tomasz Niźnikowski, Jerzy Sadowski, Andrzej Mastalerz, Jared Porter, Hubert Makaruk, Emilio Fernández-Rodríguez, Marcin Starzak, Oscar Romero-Ramos, Janusz Zieliński, Anna Bodasińska, Agata Chaliburda, Paweł Różański

**Affiliations:** 1 Faculty of Physical Education and Health in Biała Podlaska, Józef Piłsudski University of Physical Education in Warsaw, 00-968 Warsaw, Poland; jerzy.sadowski@awf.edu.pl (J.S.); hubert.makaruk@awf.edu.pl (H.M.); marcin.starzak@awf.edu.pl (M.S.); janusz.zielinski@awf.edu.pl (J.Z.); anna.bodasinska@awf.edu.pl (A.B.); agata.chaliburda@awf.edu.pl (A.C.); pawel.rozanski@awf.edu.pl (P.R.); 2 Faculty of Physical Education, Józef Piłsudski University of Physical Education in Warsaw, 00-968 Warsaw, Poland; andrzej.mastalerz@awf.edu.pl; 3 Department of Kinesiology, Recreation, and Sport Studies, The University of Tennessee, Knoxville, TN 37996, USA; jporter@utk.edu; 4 Sport Department, Faculty of Education Sciences, University of Malaga, 29071 Malaga, Spain; effernandez@uma.es (E.F.-R.); oromero@uma.es (O.R.-R.)

**Keywords:** soccer, penalty kick, OPTIMAL theory, kicking accuracy, kicking precision

## Abstract

This study investigated the immediate and cumulative effects of attentional focus (external vs. internal), practice autonomy, and their combination on soccer penalty kick performance. Methods: Ninety physically active male university students (average age 22.8 ± 1.5 years) were selected from a pool of 330 students who completed a 60 h university soccer course. Participants were randomly divided into six groups: external focus with target choice (EF-TC), external focus without target choice (EF-NTC), internal focus with target choice (IF-TC), internal focus without target choice (IF-NTC), autonomy support (AS), and a control group (C). Results: The EF-TC group demonstrated significantly higher accuracy than the IF-TC, IF-NTC, and C groups while performing comparably to the EF-NTC and AS groups in between-group analyses. Notably, the EF-NTC group showed the largest within-group improvement from pre-test to acquisition. Conclusions: The findings indicate that combining attentional focus with practice autonomy enhances the accuracy of penalty kicks, emphasizing the potential of tailored training methods for improving penalty kick performance in soccer.

## 1. Introduction

Soccer is one of the most widely practiced and globally followed team sports. Among the many technical skills required in soccer, the ability to perform an accurate penalty kick is particularly critical, especially in high-pressure scenarios. Penalty kicks often determine the results of major tournaments. For instance, Dalton [1] found that successful penalty kicks increased a team’s likelihood of winning by 34%. Given this importance, the optimization of penalty kick accuracy has garnered substantial attention from both researchers and practitioners [2,3,4,5]. One of the primary challenges faced by soccer coaches is creating practice environments that effectively improve kicking accuracy. While numerous studies have addressed the determinants of successful penalty kicking, much of the existing literature has predominantly focused on biomechanical variables [6], with comparatively less emphasis on cognitive and psychological factors influencing performance.

In this context, a growing body of evidence supports the notion that adopting an external focus of attention (on the effect of the movement, e.g., the ball or target) significantly enhances motor skill execution across various sports, including soccer, golf, and racket disciplines [7,8,9,10]. According to the “constrained action hypothesis” proposed by Wulf et al. [11], directing attention externally facilitates more automatic and efficient motor control, whereas an internal focus (on the body movements producing the action, e.g., the kicking leg) may disrupt performance by encouraging conscious movement regulation.

However, recent research has questioned the robustness of the external focus advantage, citing concerns about publication bias, small sample sizes, and methodological inconsistencies [12,13]. McKay et al. [14] argue that the reported benefits of external focus may be overestimated due to underpowered studies and statistical biases. McKay et al. [13] showed that, after correcting for publication bias using Bayesian models, the effects of external focus on performance, retention, and transfer were substantially reduced or statistically negligible. Subsequent analyses by McKay et al. [13] and Ref. [12] highlighted the influence of underpowered designs and selective reporting.

Another factor believed to facilitate motor learning is autonomy support. Several studies suggest that allowing learners to control aspects of their practice conditions enhances skill acquisition [15,16]. For example, self-controlled feedback [17,18], practice schedule adjustments [19,20], and choice in physical assistance [21] have been associated with improved motor learning. However, recent studies challenge the magnitude of these effects. Large-sample experiments [22,23,24,25] report null effects of self-controlled practice conditions on motor performance, suggesting that the benefits may be minimal or context-dependent. Furthermore, concerns about methodological rigor in self-controlled learning studies have been raised [26].

More recently, researchers have explored the combined effects of attentional focus and autonomy support on motor performance [27,28,29,30]. According to the OPTIMAL (Optimizing Performance Through Intrinsic Motivation and Attention for Learning) theory [30], external focus, autonomy support, and enhanced expectancies synergistically improve motor learning. Wulf et al. [31] demonstrated that combining an external focus with autonomy support led to superior throwing accuracy compared to either factor alone. However, inconsistencies in the literature remain. Some studies have failed to replicate these additive effects in tasks such as elite softball throwing [29], and others have found no significant benefits of combining external focus and autonomy support in penalty kicking [5]. The discrepancy in findings may be due to differences in task complexity, participant skill level, or study design [5]. Additionally, many studies investigating these effects focus on short-term performance rather than long-term learning, further complicating interpretations [31].

Given the mixed evidence for additive effects of attentional focus and autonomy in motor learning, further research is needed to clarify their combined effects. Unlike studies that focus solely on penalty kicks, the present research employs a target-kicking task designed to isolate accuracy training. This approach allows for a controlled investigation of motor learning principles while acknowledging that some aspects of a real penalty kick (e.g., goalkeeper reactions) are not captured. Based on previous findings, we hypothesize that combining autonomy with an external focus of attention will lead to the greatest improvements in kicking accuracy, whereas the condition with no choice and an internal focus will be the least effective. The objective of this study was therefore to determine whether combining attentional focus and practice autonomy improves penalty kick accuracy compared to attentional focus or autonomy alone.

## 2. Materials and Methods

### 2.1. Participants

The study sample consisted of 90 male participants, all of whom were healthy and physically active university students, with a mean age of 22.8 years (SD = 1.3). They were recruited from a cohort of 330 students who had completed a 60 h university-level soccer course which incorporated assessments of passing, kicking accuracy, and penalty kicking. As a result, they were classified as moderately skilled in penalty kick execution. Inclusion criteria required that participants (a) were male university students aged 21–25 years, (b) had no musculoskeletal injury in the six months preceding the study, (c) were physically active but not competing in organized or competitive soccer at the club level, and (d) self-reported right- or left-foot dominance.

To assess the adequacy of the sample size, a simulation-based power analysis was conducted in R. Using 1000 simulated datasets (separately for MRE and BVE), we assessed the frequency with which a significant effect would be detected under the assumption of the proposed effect size (d = 0.50) and sample size (90 participants). The simulation results indicated an estimated power of 0.513, suggesting a moderate probability of detecting a true effect. This finding provided confidence that the study design, with the specified sample size, was capable of identifying meaningful differences, while also indicating a moderate risk of Type II error (failing to detect a true effect).

Most participants were right-foot dominant (*n* = 80), while 10 identified as left-foot dominant. All individuals reported normal or corrected-to-normal vision and agreed to refrain from practicing the task or engaging in related activities outside of scheduled sessions during the study. Prior to participation, informed consent was obtained from all subjects. Participants were unaware of the specific research hypotheses to minimize expectancy effects and were informed of their right to withdraw at any time.

### 2.2. Experimental Procedure

**Figure 1:**
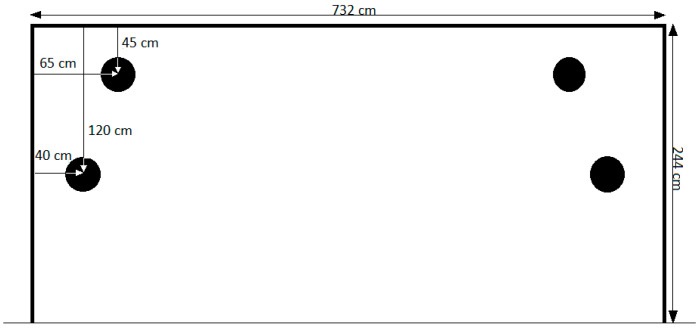
A schematic representation of the goal area and four circular targets used during the penalty kick (frontal view).

The experiment was conducted in an indoor sports facility using a simulated goal area (Figure 1).

A full-scale goal representation (7.32 m × 2.44 m) was projected onto a white wall. Four circular target zones (black circles, 30 cm in diameter) were marked on the wall to represent typical high-probability scoring areas identified in prior studies [4,32]. The soccer ball was placed at a fixed distance of 11 m away from the center of the goal, compliant with FIFA regulations (IFAB, 2016). A regular size five inflated to 9 psi soccer ball was used.

Each penalty trial was visually recorded using two 50 Hz digital cameras (Sony, TRV 900E, Tokyo, Japan) at a shutter speed of 10 kHz. Both digital cameras were positioned on the diagonal level of the floor at a distance of 12 m from the goal plane, forming an approximate 90° angle with the goal plane. One camera was mounted 3 m to the right side of the goal plane, capturing a field of view of two targets on the upper and lower edges of the goal plane. The second camera was mounted 3 m to the left of the goal plane. Screenshots extracted from the video recordings were analyzed using R software to determine the distance between the center of the target and the point of ball impact on the wall. To determine the two-dimensional kinetic data, 1 point on the ball and 1 point for every four targets on the goal plane were digitized manually using the Ariel Performance Analysis System (APAS, San Diego, CA, USA). Two-dimensional coordinates were constructed using the direct linear transformation method. A low-pass digital filter with a cutoff frequency of 4 Hz was used for the smoothing of the raw position–time data. To calibrate the viewing area in 2 dimensions, a rectangle located in the right and left corner of the goal plane with four control points of 150 cm length and 200 cm height was used. The mean reconstruction error was calculated as the difference between the real dimensions of the rectangle and those presented by the APAS system. The lengths obtained were as follows: length—1.490 ± 0.051 m and height—1.981 ± 0.062 m. The mean reconstruction error did not exceed 1%.

Kicking space accuracy was assessed using the mean radial error (MRE), and precision was quantified via the bivariate variable error (BVE) [33]. These measures represent, respectively, the average distance of kicks from the intended target and the variability in kicks around a central location. The *MRE* was calculated according to Equation (1):

(1) $$MRE=RE=\;\frac{1}{k}\sum_{l=1}^{k}{RE}_{i}$$

where *RE_i_* is the radial error for trial *i*, computed as follows:

(2) $${RE}_{i}=\;\sqrt{X_{1}^{2}+Y_{1}^{2\;\;}}$$

Here, *X_i_* and *Y_i_* represent the horizontal and vertical deviations of the ball from the center of the target, respectively, in each trial. *MRE* reflects the average radial deviation across all *k* kicks performed by a participant.

The *BVE* was computed using Equation (2):

(3) $$BVE=\;\sqrt{\frac{1}{k}}\sum_{i=1}^{k}(X_{i}-\;X_{C}{)}^{2}+(Y_{2}-\;Y_{C}{)}^{2\;}$$

where *X_C_* and *Y_C_* represent the mean horizontal and vertical coordinates of all kicks, and *X_i_*, *X_i_* denote the coordinates for each individual kick. *BVE* thus quantifies the consistency of kick placement.

All penalty kicks that struck the wall within the camera’s calibrated frame were included in the analysis. For any trial in which the ball fell outside the field of view (i.e., more than 2.5 m from the target center), a fixed error value of 2.5 m was assigned. Notably, 97% of the recorded kicks remained within the measurement area.

### 2.3. Design and Intervention

The experiment consisted of three phases: a pre-test (PreT), acquisition test (AT), and retention effects, conducted two days after the acquisition period. The experiment was conducted over four weeks. Before the beginning of the experiment, participants were familiarized with the aim of the study without being provided knowledge regarding the expected effects (outcomes) of different models of attentional focus or autonomy support on kicking performance. All participants were given basic instructions regarding how to execute the penalty kick. The basic instructions were followed by one demonstration performed by an expert soccer player. Participants were asked to duplicate the task to hit the target as accurately as possible. After an initial familiarization session, participants performed a 12-trial pre-test, with three kicks directed toward each target. All attempts were performed using the dominant leg, and no target was repeated in consecutive trials Participants were allowed to run up and kick the ball. The acquisition period comprised seven non-consecutive practice sessions, each consisting of 12 penalty kicks, with 84 trials per participant in total.

**Figure 2:**
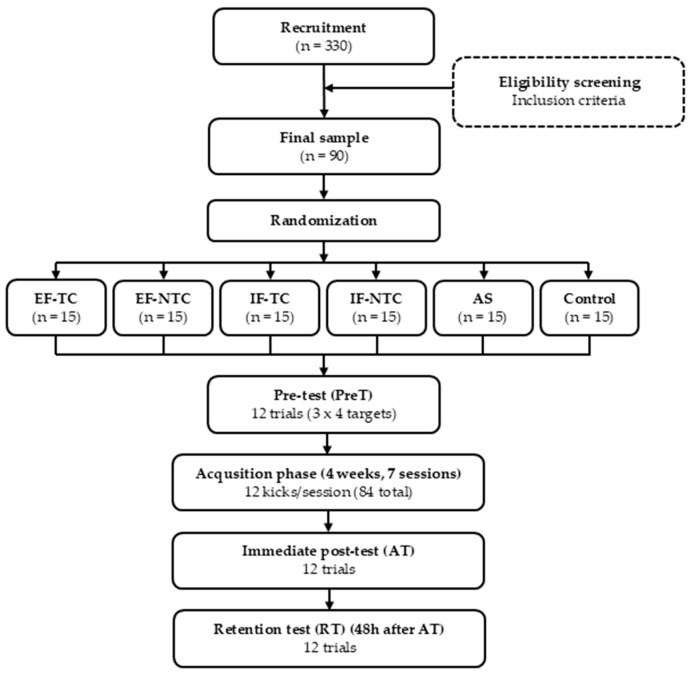
Flow chart of experimental design.

Prior to commencing the training session, participants were randomly assigned to one of six experimental groups, defined by attentional focus (external vs. internal) and level of autonomy (choice vs. no choice): external focus with target choice (EF-TC), external focus without target choice (EF-NTC), internal focus with target choice (IF-TC), IF- internal focus without target choice (NTC), autonomy support with target choice (AS), and control group, which did not participate in the intervention and performed only pre-tests and an acquisition test (C). Participants in the IF-NTC and IF-C groups were instructed to focus their attention on the movement of their kicking leg, while those in the EF-NTC and EF-TC groups were asked to concentrate on the given target. Participants in the AS and C groups were not given any specific focus of attention instructions. Moreover, the IF-TC and EF-TC groups were informed that they could choose the target sequence they wanted to kick the ball toward; however, they were not allowed to do so consecutively to the same target. The order of kicking the ball for the EF-NTC and IF-NTC groups was randomized. Participants in the AS group were instructed to select the sequence of targets and verbally inform the experimenter about their intended target before taking each shot (e.g., “I choose to kick the ball to the top target on the right side”). For the EF-NTC and IF-NTC groups, the experimenter announced the target location before each attempt (side and height) to standardize goal location across trials. This announcement was not feedback about the result of any previous attempt. A schematic overview of the study design is presented in Figure 2.

All participants were informed that they would only use their dominant leg and were instructed before the first, fourth, and eighth trials to perform the task to the best of their ability. Additionally, participants in groups EF-NTC and IF-NTC were verbally informed of the location of each shoot (i.e., side and height). No feedback was provided to the participants other than inherent feedback that was visually available to them by performing the penalty kick.

Before each session, all participants completed a standardized 10 min warm-up, consisting of jogging and dynamic stretching. Participants were asked to wear standardized footwear to reduce equipment-based performance variance. An immediate retention test was administered after the final practice session on day four. The retention test was assessed two days later by an acquisition test. The pre-test, acquisition test, and retention tests consisted of 12 trials, each with four attempts at each target, with no consecutive trials occurring at the same target.

### 2.4. Data Analyses

All statistical analyses were conducted in R (version 4.4.3, Windows 11 x64) using the lme4 and emmeans packages. Prior to model estimation, diagnostic checks were performed focusing on visual inspection of residuals rather than formal normality tests, as the latter add little in the context of mixed-effects models. Specifically, Q–Q plots and residual fitted value plots were used to assess the approximate normality and homoscedasticity of residuals. No severe deviations were detected. Importantly, in repeated-measures designs, the dependence of observations is expected and explicitly modelled via random effects rather than treated as a violated assumption.

The primary analysis employed a linear mixed-effects model (lmer) with Time (PreT, AT, RT), Group (EF-TC, EF-NTC, IF-TC, IF-NTC, AS, C), and their Group × Time interaction as fixed effects. A random intercept for each participant (ID) was included to account for between-subject heterogeneity and repeated measures over time. The model was estimated using restricted maximum likelihood (REML) to obtain unbiased variance components.

The inclusion of the Group × Time interaction was essential, as without it one cannot test for differential improvements across groups. Estimated marginal means with Bonferroni-adjusted post hoc comparisons were reported to aid interpretation, but emphasis was placed on model-based main and interaction effects rather than isolated pairwise contrasts.

Regarding statistical power, a priori simulations for a moderate effect size (d = 0.50) yielded power = 0.51 for the six-arm, three-time-point design. We acknowledge that this reflects low power for small to moderate effects, meaning null results should be interpreted cautiously. Detecting smaller effects would require substantially larger samples; however, the present analysis aimed to provide initial evidence rather than definitive conclusions.

Effect sizes (Cohen’s d) were computed from the estimated marginal means for descriptive purposes, using thresholds of small (d < 0.5), moderate (0.5 ≤ d < 0.8), and large (d ≥ 0.8) effects [34].

## 3. Results

A mixed linear model (lmer) with fixed effects for Time, Group, and their interaction (Group × Time) and a random intercept for each participant was used to analyze the data, with REML estimation providing unbiased variance components. This approach accounts for between-group differences and repeated measures within participants across the three time points: pre-test, acquisition test (AT), and retention test (RT). Including participant ID as a random intercept accounted for within-subject dependence and individual-level heterogeneity, ensuring appropriate variance partitioning and preventing inflated Type I error rates. Primary inferences were based on the full model estimates for Time, Group, and Group × Time interaction terms, while post hoc comparisons using emmeans with Bonferroni correction were used only to illustrate specific contrasts, acknowledging reduced statistical power due to multiple testing and the study’s moderate sample size.

Data analysis was based on the three measurement points—pre-test, acquisition test (AT), and retention test (RT) for both MRE and BVE across six groups: EF-TC (external focus with target choice), EF-NTC (external focus without target choice), IF-TC (internal focus with target choice), IF-NTC (internal focus without target choice), AS (autonomy support with target choice), and C (control group, which did not participate in the intervention and performed only pre-tests and an acquisition test). Detailed results from the analysis are presented below. Fixed-effect estimates (β, SE, 95% CI, *p*) for all model terms, as well as variance components, are summarized in Table 1 and Table 2. This approach emphasizes model-based inference with interaction terms rather than isolated pointwise tests, ensuring valid interpretation of Group × Time effects.

**Kicking Accuracy (MRE)**

The mixed-effects model revealed a significant main effect of Time (AT vs. PreT: β = −9.87, SE = 3.12, 95% CI [−15.92, −3.82], *p* = 0.002), indicating that kicking accuracy improved after training across all groups (Table 1). However, significant Group × Time interactions clarified that improvement trajectories differed by condition, highlighting that main effects alone cannot capture group-specific intervention responses.

Specifically, the EF-NTC group at AT showed the largest reduction in MRE relative to the control group (β = −11.41, SE = 4.35, 95% CI [−19.95, −2.87], *p* = 0.009), whereas the IF-NTC group at RT exhibited the highest MRE (β = 8.97, SE = 4.11, 95% CI [1.01, 16.93], *p* = 0.027), indicating less training benefit and weaker retention effects.

Variance components indicated substantial between-participant heterogeneity (SD = 6.92) relative to residual error (SD = 4.81), confirming the need for a random intercept to account for individual-level variability.

Combined within- and between-group comparisons showed that the EF-NTC group demonstrated significant improvement in accuracy from PreT to AT (*p* = 0.0132; d = 0.714), while the EF-TC group showed a significantly lower MRE than the IF-NTC group at both PreT (*p* = 0.0163; d = 1.73) and RT (*p* = 0.0010; d = 1.79). At retention, the IF-NTC group again exhibited the highest MRE (59.8 ± 18.6), significantly worse than the AS group’s result at acquisition (*p* = 0.0294, d = 1.16). The control group (C) showed stable accuracy across all time points (Table 2).

**Kicking Precision (BVE)**

Similarly, Time showed a significant main effect (AT vs. PreT: β = −7.53, SE = 2.98, 95% CI [−13.38, −1.68], *p* = 0.012), reflecting short-term improvement in precision after training (Table 3. However, Group × Time interactions clarified that precision changes were group-dependent, again emphasizing that main effects alone cannot be interpreted in isolation.

The EF-TC group at AT had a significantly lower BVE compared to the control group (β = −9.24, SE = 3.97, 95% CI [−16.95, −1.53], *p* = 0.019), whereas its RT performance was descriptively higher but non-significant (β = 6.11, SE = 4.21, 95% CI [−2.13, 14.35], *p* = 0.143), indicating a lack of retention for precision improvements.

Variance estimates revealed that the random-intercept SD (5.34) exceeded the residual SD (3.92), highlighting meaningful participant-level heterogeneity and supporting the inclusion of a multilevel structure in the model.

Combined within- and between-group comparisons indicated that although both EF-TC and EF-NTC groups showed reductions in BVE following the intervention, only the EF-TC group achieved a significantly lower BVE than the IF-NTC group at retention (*p* = 0.0203; d = 1.17). By RT, the EF-TC group exhibited the highest BVE (48.6 ± 16.5), whereas the EF-NTC (36.6 ± 12.5) and EF-TC (34.8 ± 13.2) groups maintained lower BVE scores than the control group (45.1 ± 9.2), although these differences were not statistically significant (Table 4).

### Summary of Effects

Taken together, these results indicate that accuracy (MRE) improved most in the EF-NTC group, whereas precision (BVE) improvements were observed only in the EF-TC group and were not maintained at the retention test (RT). Significant Group × Time interactions confirmed that training effects varied across groups and time points, while variance components highlighted substantial individual-level differences beyond residual error. In terms of accuracy, the EF-NTC group demonstrated the greatest improvement, whereas the IF-NTC group consistently showed the highest error levels across time points, indicating the weakest intervention effect. For precision, the EF-TC group exhibited short-term gains compared to the IF-NTC group, but these improvements did not generalize across all groups or persist at RT, suggesting limited retention of precision benefits.

## 4. Discussion

The present study aimed to examine the effects of attentional focus and autonomy support on penalty kick performance in moderately skilled soccer players. It was hypothesized that external focus combined with autonomy support would yield superior penalty kick accuracy and precision, whereas internal focus without autonomy would result in the weakest performance.

The results partially confirmed these hypotheses. The external focus with choice group showed a significant improvement in accuracy compared to the internal focus without choice group in both the acquisition and retention phases, indicating that external attentional cues alone were effective in facilitating short-term performance gains. The absence of improvement in the external focus without choice group suggests that external focus alone may be insufficient, and that autonomy support potentially plays a facilitative role in learning retention. This pattern indicates that combining external focus and autonomy support may support learning retention over time, even if immediate benefits are not pronounced.

In contrast, the internal focus with no-choice group consistently demonstrated the lowest performance across all time points, which supports prior evidence that internal focus impairs automatic control processes and increases movement inefficiency [7]. According to the “constrained action hypothesis” [11], internally directed attention induces conscious control over movements, thereby disrupting the automaticity needed for fluent execution. Conversely, an external focus is thought to facilitate automaticity by directing attention toward the intended effect of the movement, allowing the motor system to self-organize with reduced conscious involvement [35]. These mechanisms may explain why the external focus with choice group improved more rapidly, particularly in early phases of skill acquisition where attentional load and execution demands remain high [36,37].

The additive benefit of autonomy support, hypothesized to enhance learning through motivational and cognitive pathways [15,16], was partially supported by the results. Although the external focus with choice group outperformed the internal focus without choice group and showed the most favorable descriptive improvement in the retention test, these effects were not statistically significant in all conditions. This contrast was particularly evident when comparing the external focus with choice group to the autonomy-only group, suggesting that attentional cues may have played a more substantial role than choice alone. Prior work has suggested that allowing learners to make choices can support deeper processing and greater self-regulation [38,39], and enhance motivation by fulfilling the psychological need for autonomy [40]. However, recent studies have cast doubt on the robustness of these effects. For instance, Bacelar et al. [22] reported that while autonomy-supportive practice increased perceived autonomy, it did not consistently improve motor learning outcomes. Moreover, McKay & Ste-Marie [23] and St. Germain et al. [24,25] found limited or null effects of autonomy support in larger, well-controlled trials. These findings imply that the effectiveness of autonomy support is not universal but likely depends on specific contextual variables, including task complexity, the learner’s prior experience, and the way autonomy is operationalized within the practice environment. Importantly, no significant interaction between attentional focus and autonomy support was observed across time points, indicating that the effects of these variables were primarily additive rather than synergistic.

The broader efficacy of external focus has also been questioned. McKay et al. [14] demonstrated that after correcting for reporting bias using robust Bayesian modeling, the average effects of external focus on performance, retention, and transfer were substantially attenuated. These findings suggest that much of the previously reported advantage of external focus may have been overestimated due to selective reporting and underpowered studies [13,41]. Taken together, this emerging body of work supports a more cautious interpretation of attentional and autonomy effects, emphasizing the need for greater methodological precision in future studies.

In terms of kicking precision, no statistically significant within-group improvements were observed over time. However, a significant between-group difference emerged during the retention test. The external focus with target choice group demonstrated lower precision scores (the ball was directed closer to the target) than the internal focus without choice group, indicating greater consistency of motor output in the former condition. These findings align with prior studies indicating that external focus effects may emerge gradually and be more evident in retention or transfer phases [26,42], which is consistent with the delayed pattern observed in the external focus with choice group. These findings align with prior studies [26,42] that highlight how the benefits of attentional focus may emerge more clearly over time, supporting the notion that delayed retention measures better capture true learning effects. The absence of statistically significant effects on precision in the present study may also reflect limitations in the sensitivity of the measurement approach or task-specific factors that constrain the detection of learning-related changes beyond directional accuracy.

Despite the widespread belief that external attention and autonomy support practice are beneficial for enhancing motor learning or performance, researchers continue to debate the underlying mechanisms responsible for these effects [43]. The study conducted by Wulf et al. [31] yielded results that are broadly consistent with the present findings. In their experiment, combining external focus with autonomy support resulted in better throwing accuracy on both retention and transfer tests than either condition alone. A possible explanation for the partial convergence between our results and theirs may lie in differences in sample characteristics and task design. While Wulf et al. [31] examined novice learners performing a novel skill, the current study involved moderately skilled soccer players with prior experience (~60 h of practice) executing a familiar but high-demand task. The greater baseline proficiency of our participants may have moderated the effects of autonomy, which often show stronger effects in early skill acquisition stages. These differences highlight the importance of task complexity and learner experience in determining the impact of attentional and motivational interventions.

Although these findings generally align with previous work demonstrating the advantages of external focus and learner autonomy [15,30], they also reinforce the idea previously observed by Makaruk et al. [5] that these benefits are not always additive. This was evident in our results, where autonomy support did not enhance the effects of external focus during acquisition, and only modestly during retention. These findings reflect a pattern observed in other sport-specific studies, suggesting that the effectiveness of combining autonomy and attentional focus may depend on contextual variables such as task complexity and learner experience.

Several limitations should be acknowledged. The absence of a manipulation check limits certainty that participants adhered to attentional focus instructions throughout all trials. Consequently, sustained adherence to the assigned focus cannot be verified. We mitigated this risk through standardized scripting, delivery at consistent time points, and minimizing experimenter–participant interaction during trials; nonetheless, we recognize this as a limitation. Additionally, the retention interval was relatively short, capturing only immediate consolidation effects rather than long-term retention. The penalty kick task, while ecologically relevant, lacked goalkeeper presence or competitive pressure, which may influence attentional demands and learner engagement. Furthermore, the study included only male students, which limits the generalizability of the findings across genders. While the sample size was sufficient to detect moderate effects, it may have been insufficient to detect smaller or interaction effects between attentional focus and autonomy. In addition, the training period was relatively short, and future studies should extend the intervention to better capture long-term learning effects. Because choice groups could select targets (with no consecutive repetitions), potential target preference or sequencing effects may have influenced performance. Although MRE and BVE are robust to within-movement variability, unpredictable motor adjustments could still add noise. Future studies should control for this by recording target identity, balancing sequences, or stratifying randomization.

In summary, the present study examined the isolated and combined effects of attentional focus and autonomy support on the accuracy and precision of soccer penalty kicks among moderately skilled performers. The results provide partial support for the proposed hypotheses. The external focus with choice group demonstrated the most substantial improvement in both kicking accuracy and consistency relative to the internal focus without choice group, highlighting the effectiveness of combining external focus and autonomy. While autonomy support did not significantly enhance external focus effects across all measures, the trend in both kicking accuracy and precision indicates that learner-directed practice may facilitate longer-term performance benefits.

## 5. Conclusions

These findings contribute to the growing literature on the mechanisms underlying effective motor learning and highlight the need for nuanced interpretation of both attentional and motivational factors. In particular, the results underscore the importance of attentional focus as a performance-enhancing strategy. Both internal and external focus conditions improved penalty kick accuracy in the short term, but only external focus demonstrated superior retention effects. Thus, adopting some form of attentional focus is advantageous, with external focus emerging as the more effective strategy for sustaining accuracy over time. However, given the variability in effects across tasks and populations, autonomy support may provide additional benefits under specific conditions and should be considered with regard to context, athlete characteristics, and training goals.

From an applied perspective, coaches and practitioners may consider incorporating externally focused instructions during motor skill acquisition. While granting athletes autonomy may offer motivational benefits, its influence on learning appears to depend on factors such as task complexity and athlete experience. Future research should identify the most effective forms of external focus and autonomy support for complex motor tasks across different skill levels and sport-specific contexts. In addition, future research should examine younger populations, particularly adolescent athletes, to assess whether the benefits observed in university students generalize to earlier stages of skill acquisition.

## Figures and Tables

**Table 1:** Fixed-effect estimates for MRE.

Effect	Beta	SE	CI Lower	CI Upper	*p*-Value
Intercept (C, PreT)	51.054	3.491	44.212	57.897	**0.000**
Time (AS vs. PreT)	0.452	4.322	−8.020	8.923	0.917
Time (RT vs. PreT)	0.402	4.322	−8.069	8.873	0.926
Group (AS)	2.486	4.937	−7.191	12.163	0.615
Group (EF-NTC)	1.657	4.937	−8.020	11.334	0.737
Group (EF-TC)	0.367	4.937	−9.310	10.044	0.941
Group (IF-NTC)	5.578	4.937	−4.099	15.255	0.259
Group (IF-TC)	3.331	4.937	−6.346	13.008	0.500
Group × Time (AS × AT)	−12.113	6.112	−24.093	−0.133	**0.048**
Group × Time (AS × RT)	−2.153	6.112	−14.133	9.827	0.725
Group × Time (EF-NTC × AT)	−9.564	6.112	−21.544	2.416	0.118
Group × Time (EF-NTC × RT)	−8.422	6.112	−20.402	3.558	0.168
Group × Time (EF-TC × AT)	−13.846	6.112	−25.826	−1.866	**0.023**
Group × Time (EF-TC × RT)	−8.728	6.112	−20.708	3.252	0.153
Group × Time (IF-NTC × AT)	−6.791	6.112	−18.771	5.189	0.267
Group × Time (IF-NTC × RT)	2.729	6.112	−9.251	14.709	0.655
Group × Time (IF-TC × AT)	−7.076	6.112	−19.056	4.904	0.247
Group × Time (IF-TC × RT)	−0.252	6.112	−12.232	11.728	0.967
Group Var	0.305	0.121	0.069	0.541	**0.011**

Note: Intercept = mean for the control group (C) at the pre-test (PreT) time point. Bold *p*-values indicate statistical significance at *p* < 0.05.

**Table 2:** MRE analysis results for groups (EF-TC, EF-NTC, AS, IF-TC, IF-NTC and C) and time-course measurements (PreT, AT, RT).

Group	PreT (Mean ± SD)	AT (Mean ± SD)	RT (Mean ± SD)	Post Hoc Comparisons	*p*-Value (Post Hoc)
EF-TC	51.4 ± 12.5	38.0 ± 10.0	43.1 ± 16.1	EF-TC (AT) < IF-NTC (PreT) EF-TC (AT) < IF-NTC (RT)	*p* = 0.0163 *p* = 0.0010
EF-NTC	52.7 ± 18.2	43.6 ± 12.6	44.7 ± 12.9		
AS	53.5 ± 15.8	41.9 ± 16.7	51.8 ± 16.3	AS (AT) < IF-NTC (RT)	*p* = 0.0294
IF-TC	54.4 ± 11.2	47.8 ± 14.7	54.5 ± 12.3		
IF-NTC	56.6 ± 11.4	50.3 ± 14.1	59.8 ± 18.6		
C	51.1 ± 6.1	51.5 ± 7.2	51.5 ± 8.5		

Note: EF-TC = external focus with target choice; EF-NTC = external focus without target choice; IF-TC = internal focus with target choice; IF-NTC = internal focus without target choice; AS = autonomy support; C = control; MRE = mean radial error.

**Table 3:** Fixed-effect estimates for BVE.

Effect	Beta	SE	CI Lower	CI Upper	*p*-Value
Intercept (C, PreT)	43.846	2.951	38.061	49.630	**0.000**
Time (AT vs. PreT)	1.214	3.697	−6.032	8.459	0.743
Time (RT vs. PreT)	1.217	3.697	−6.028	8.463	0.742
Group (AS)	0.218	4.174	−7.963	8.399	0.958
Group (EF-NTC)	−2.670	4.174	−10.851	5.511	0.522
Group (EF-TC)	−1.658	4.174	−9.839	6.523	0.691
Group (IF-NTC)	3.181	4.174	−5.000	11.362	0.446
Group (IF-TC)	0.872	4.174	−7.309	9.053	0.834
Group × Time (AS × AT)	−11.515	5.228	−21.762	−1.268	**0.028**
Group × Time (AS × RT)	−2.325	5.228	−12.572	7.922	0.657
Group × Time (EF-NTC × AT)	−3.681	5.228	−13.928	6.566	0.481
Group × Time (EF-NTC × RT)	−5.757	5.228	−16.004	4.489	0.271
Group × Time (EF-TC × AT)	−10.763	5.228	−21.010	−0.517	**0.040**
Group × Time (EF-TC × RT)	−8.619	5.228	−18.866	1.627	0.099
Group × Time (IF-NTC × AT)	−7.304	5.228	−17.550	2.943	0.162
Group × Time (IF-NTC × RT)	0.316	5.228	−9.931	10.563	0.952
Group × Time (IF-TC × AT)	−7.607	5.228	−17.854	2.639	0.146
Group × Time (IF-TC × RT)	−3.258	5.228	−13.504	6.989	0.533
Group Var	0.275	0.115	0.050	0.500	**0.017**

Note: Intercept = mean for the control group (C) at the pre-test (PreT) time point. Bold *p*-values indicate statistical significance at *p* < 0.05.

**Table 4:** BVE analysis results for groups (EF-TC, EF-NTC, AS, IF-TC, IF-NTC, and C) and time-course measurements (PreT, AT, RT).

Group	PreT (Mean ± SD)	AT (Mean ± SD)	RT (Mean ± SD)	Post Hoc Comparisons	*p*-Value (Post Hoc)
EF-TC	42.2 ± 12.1	32.6 ± 10.0	34.8 ± 13.2	EF-TC (AT) < IF-NTC (RT)	*p* = 0.0203
EF-NTC	41.2 ± 14.8	38.7 ± 12.9	36.6 ± 12.5		
AS	44.1 ± 14.5	33.8 ± 12.2	43.0 ± 15.7	AS (AT) < IF-NTC (RT)	*p* = 0.0570
IF-TC	44.7 ± 12.3	38.3 ± 12.5	42.7 ± 14.8		
IF-NTC	47.0 ± 11.4	40.9 ± 13.0	48.6 ± 16.5		
C	43.8 ± 9.7	45.1 ± 8.5	45.1 ± 9.2		

Note: EF-TC = external focus with target choice; EF-NTC = external focus without target choice; IF-TC = internal focus with target choice; IF-NTC = internal focus without target choice; AS = autonomy support; C = control; BVE = bivariate variable error.

## Data Availability

The data are available at https://osf.io/xusw3 (accessed on 12 August 2025).
